# Editorial: When (and how) Theory of Mind is useful? Evidence from innovative assessment tools, training, and treatments strategies, volume II

**DOI:** 10.3389/fpsyg.2025.1721691

**Published:** 2025-11-18

**Authors:** Francesca Baglio, Sara Isernia, Simone Shamay-Tsoory, Antonella Marchetti

**Affiliations:** 1 IRCCS Fondazione Don Carlo Gnocchi ONLUS, Milan, Italy; 2 Department of Psychology, University of Haifa, Haifa, Israel; 3 Research Unit on Theory of Mind, Department of Psychology, Università Cattolica del Sacro Cuore, Milan, Italy

**Keywords:** theory of mind, social cognition, assessment, treatment, rehabilitation, mentalizing

Social cognition (SC) refers to several core competencies that allow individuals to successfully adapt to the interpersonal world, managing others' emotions, thoughts, and behaviors, which is essential for daily interactions and overall wellbeing (Arioli et al., 2018). Despite its importance, it has long been neglected in clinical settings, which have focused more on traditional cognitive functions. Only with the Diagnostic and Statistical Manual of Mental Disorders, Fifth Edition (DSM-5) SC was recognized as a core neurocognitive domain (Sachdev et al., 2014). This late acknowledgment has resulted in a lack of standardized assessment tools, limiting both diagnostic accuracy and targeted intervention development (Cerami et al., 2025). This Research Topic addresses that gap, focusing on Theory of Mind (ToM), a core component of SC defined as the capacity to understand and predict behavior based on one's own and others' mental states. Building on the foundation laid by our earlier volume (Baglio and Marchetti, 2016), this Research Topic advances the field by presenting studies that develop effective assessment tools and propose innovative training strategies for therapeutic interventions. Overall the Research Topic brings together nine contributions from 44 internationally recognized authors, including seven original research articles, one perspective piece, and one opinion article. Collectively, their studies focus on developmental stages of ToM and on the research of effective tools for its assessment, as well as strategies aimed at enhancing it. Erceg et al. present a longitudinal framework of ToM measurement across the lifespan. The authors identify a critical developmental window between ages 6 and 9, which differs from adulthood, with ToM components remaining generally stable after age 9. Other studies concentrate on childhood and adolescence, stages of life in which ToM undergoes particularly significant changes. Bianco et al. address a key gap: while first-order reasoning is well-studied, second-order reasoning is less charted. Using a novel narrative-based paradigm, the authors elicited children's reasoning about characters' beliefs and desires, manipulating both valence and truth-value. Their findings reveal that second-order reasoning is more robust when associated with positive desires and true beliefs, providing important new insights into the cognitive mechanisms underpinning ToM development. Cornaggia et al. extend this research trajectory into adolescence by exploring the relationship between ToM and metalinguistic competences. Using a comprehensive assessment battery, they investigate how definitional skills and ToM interact as manifestations of broader metarepresentational abilities. Their results suggest a complex interplay: ToM performance appears to predict, albeit modestly, the ability to define ToM-related words, reinforcing long-standing evidence for a close connection between language and SC. The identification of atypical mentalizing patterns remains a central challenge in the field, particularly in the context of clinical conditions. Sharp et al. address this by introducing a novel self-report measure of hypermentalizing—where individuals draw unwarranted inferences about others' mental states. The tool is novel for being patient-reported and grounded in attachment-based theories, which allow for the consideration of different levels of everyday social relationships. The authors demonstrate the clinical utility of the tool by testing it with adolescents with personality disorders, offering a tool to identify maladaptive social cognition. Fadda et al. offer another significant advance by applying the Theory of Mind Assessment Scale (Th.o.m.a.s.) to adolescents with autism spectrum disorder. This semi-structured interview captures multiple dimensions of ToM, including Awareness (perceiving mental states in self and others), Relation (understanding causal links between mental states and behaviors), and Realization (adopting strategies to attain a desired state). Their work is enriched by the complementary review of Gabbatore et al., who critically appraise the strengths and weaknesses of widely used ToM measures. They argue that Th.o.m.a.s. is a promising tool for assessing mentalizing. At the other end of the lifespan, Sola et al. examine SC in the context of aging and neurodegenerative disorders. Using an assessment battery targeting both basic emotion recognition and affective ToM, they compare healthy older adults with individuals affected by frontotemporal dementia and Alzheimer's disease. Their findings demonstrate that frontotemporal dementia is associated with profound deficits across both domains of SC, in contrast to the comparatively milder difficulties observed in Alzheimer's disease. These results contribute to refining the clinical characterization of neurodegenerative profiles and highlight the potential diagnostic value of SC measures in differentiating neurodegenerative conditions. While assessment is crucial, translating insights into effective interventions is equally important. Two contributions address this challenge directly. Grazzani presents the PROMEHS program, which uses linguistic-conversational training activities with preschool children. Through shared story-reading, film viewing, and group discussion, the program encourages children to explore characters' inner worlds, promoting perspective-taking, emotion recognition, and the linking of emotions with behaviors. Preliminary findings suggest that this approach can foster key social competencies at an early stage, laying a foundation for lifelong social and emotional health. Birch et al. turn their attention to adolescence and adulthood, examining how interventions can address social cognitive biases as mechanisms of change. By focusing explicitly on reducing biases in social information processing, they argue that SC training can achieve more generalizable benefits, enhancing emotional wellbeing and relationships. Taken together, these contributions enrich the field of SC by advancing both theoretical and methodological perspectives. It is interesting to note that, across the studies presented here, existing measures largely remain anchored in traditional methodologies. In the future, it will be important to investigate how emerging technologies and innovative tools, such as virtual reality, can create immersive, ecologically valid scenarios that mirror real-life social interactions. Similarly, real-world (second-person) and digital phenotyping approaches may offer dynamic and fine-grained insights into brain and behavior within naturalistic contexts. Integrating these technologies into research and clinical practice represents a promising step forward—one that could shift the field from measurement toward meaningful impact, fostering interventions that are both effective and sensitive to lived experience. It is our hope that the insights offered in this Research Topic will stimulate further research, encourage cross-disciplinary dialogue, and inspire new directions in understanding SC across the lifespan.
